# Supplementary material for: Exploring the content of the STAND-VR intervention: A qualitative interview study
Source: PLOS Digit Health. 2023 Mar 13;2(3):e0000210. doi: 10.1371/journal.pdig.0000210 (PMC10010507; doi:10.1371/journal.pdig.0000210)
Supplement: S1 Text — (DOCX) [file pdig.0000210.s001.docx]

**Interview Schedule**

**PURPOSE**

- The purpose of this interview is to get your opinion.
- Your contribution will help inform the progress of this PhD project.
- The purpose of my PhD is to encourage retired and non-working adults over the age of 55 to reduce their time spent sitting each waking day by adopting meaningful non-sedentary activities in virtual reality (**explain sedentary behaviour term**).
  - We specifically want to reduce prolonged sedentary behaviour which is about 6+ hours of sedentary activity each day and has been associated with long-term negative health outcomes.
  - Virtual reality is a way of experiencing digital (explain) environments as if you are really there.
    - ***<Back and forth discussion to tease out understanding of virtual reality>***
      - Compare to watching chat show AT HOME **vs** IN THE AUDIENCE.

| **Applications for Virtual Reality** |
| --- |
| Travel and exploration (google earth – see the pyramids of Egypt or the Great Wall of China) |
| Social connection (you can link up with your friends in VR in a setting of your choosing) |
| Entertainment (any activity you enjoy in the real world can be created in VR) |
| Exercise (light physical activity games that you enjoy) |
| Education (give examples of delivery of learning experiences – e.g., a tour of the Eifel tower and its history) |
| Games (ping pong, golf, dance, etc.) |
| Reminiscence (traveling to a place of significance to you to reminisce over your time spent there) |

- *Read scenario…*
- Developing a support system – adopt meaningful non-sedentary activities in virtual reality.
- Support system will be in virtual reality *<point at equipment>*.
  - Will be like using an application on any other digital device.
- Designed using theory (an idea a researcher has about the way something might work), scientific evidence and retired/non-working adults’ voices.
- *It is really important to note that* using virtual reality in this way is not about replacing activities in your daily life but more about enhancing and supporting the activities you already do to provide more opportunity that may help you to reduce your time spent sitting.
- A research assistant will come in briefly during the VR experience just to observe that you are doing ok and do not need any additional assistance while I am looking at what you are doing in the VE.

**INTERVIEW OUTLINE**

There are 3 separate parts to this interview. The first will be short; I will ask you a few questions about if you’ve had any experiences with digital technology in general. Part two of the interview will be the most interesting for you – I hope! I will explain how to use virtual reality and you will get to use virtual reality equipment and actually experience virtual reality first-hand. The final part of the interview is the most important for me – I will ask you questions about what you think about virtual reality, I will ask you for ideas about how virtual reality could help you spend less time sitting and I’ll share with you my ideas to see what you think of them. The goal of this interview is to get your input to help me make a meaningful virtual reality experience, so the more honest you are the better! Does that sound ok to you? OK, let’s get started!

**PART I: WARM UP**

1. Have you ever used virtual reality?
   1. **Conditional:** How did you find that experience?
2. Have you ever used any kind of digital technology to help you manage your health? (e.g., an activity watch, a mobile phone app that reminds you to move, etc.)
   1. **Prompt:** Do you have any thoughts on using technology to help you with your health?
3. Given what you know so far about virtual reality and sedentary behaviour, what are your first impressions of using virtual reality to reduce sedentary behaviour?
   1. **Prompt:** Could you see ways virtual reality could help you reduce your sedentary behaviour?

**PART II: VIRTUAL REALITY EXPERIENCE**

- Not the support system that will help you reduce your sedentary behaviour. Not created yet.
- Just a way to experience virtual reality for the first time
- Will complete some basic tasks that will allow you to interact with the virtual environment.
  - **Look** around the virtual environment to find bearings.
  - **Move** around the virtual environment.
  - **Pick up** objects in the virtual environment.
  - **Watch** a short video clip on a television in the virtual environment.
  - Also have option to see a **realistic living space** to provide a little more insight.
- I will talk you through all of it and you can stop at any time.

*Virtual reality experience (explore training environment) …*

**POST-VR EXPERIENCE QUESTIONS**

1. Tell me your first impressions of the virtual experience?
   1. **Prompt:** Can you explain how being in the virtual environment made you feel?
   2. **Prompt:** Did you feel present in the environment?
      1. How did you find that experience?
   3. **Prompt:** Do you have any thoughts on the way you were represented in the virtual environment (i.e., your hands)?
2. How did you find learning how to use the equipment to interact with the virtual environment?
   1. **Prompt:** Did you find there was anything that made it easier or more difficult to use the virtual reality equipment or virtual objects in the environment (like the blocks or the ball)?
3. Did you find the face mask affected your experience?
   1. **Conditional:** How did it affect you?
4. Any additional feedback about the virtual experience in general?

**Part III: INTERVENTION IDEA GENERATION AND DISCUSSION WARM UP**

- Next, will discuss the support system. I have identified key factors that have been found to reduce retired/non-working adults time spent sedentary. I will ask you two questions about each of these.
  - You suggest ideas as to how each of these factors could be presented in VR.
  - I will describe ideas I have about presenting these factors in VR. You can let me know what you think about them and if you find them acceptable. We can talk about how/if both of our ideas could be included then.
    - *Important note.* When thinking about virtual reality, I think the best way to imagine it is as an extension of real life. So, almost anything you can do in real life, can be created in virtual reality.
- Finally, I will ask you a few general questions at the end relating to certain preferences to be included in the virtual environment.

**INTERVENTION IDEA GENERATION AND DISCUSSION**

The following factors will be included in a support system I have created called the STAND-VR intervention.. We would appreciate your honest feedback on everything here as none of our own ideas are set in stone (**N.B.** Bear in mind that you can **disagree**, answer **no**, or answer **I don’t know** to any of these).

| **Q** | **This is what we want to encourage/facilitate - how do you think we could do that through immersive virtual reality?** | **This is what we are thinking of doing - what do you think of that?** |
| --- | --- | --- |
|  | Thinking about your experience using the virtual reality equipment, how would you like to be introduced to this equipment and learn how to use it? | - **Step-by-step guide** to the virtual reality equipment.   - E.g., Written instructions helping you to set up the virtual reality equipment for the first time.   - E.g., Have a person present to help you set up the virtual reality equipment for the first time (**who?**). |
|  | Thinking about your experience engaging with virtual environments, how would you like to be introduced to these environments and learn how to engage with them (e.g., the shapes you grabbed during your virtual experience)? | - **Step-by-step guide** to virtual environments and the STAND-VR intervention.   - E.g., A video of a person, a virtual person or written information helping you engage with virtual environments for the first time.   - E.g., Have a person present to help you engage with the virtual environments for the first time (**who?**). |
|  | Thinking about what you have learned about virtual reality today, can you think of ways you could learn about sedentary behaviour in virtual reality? | - A **reliable source** (**explore**) providing information on what sedentary behaviour is and how it differs from physical activity and physical inactivity.   - E.g., A video of a person, a virtual person or written information providing this information in virtual reality. |
|  | Can you think of strategies you could use to help you remember to take part in non-sedentary activities in virtual reality? | - **Scheduled** virtual reality activities. Send a reminder to your phone or write on a calendar to remind you to take part.   - E.g., A video of a person, a virtual person or written information helping you to use virtual reality to set up and use this feature.   - Have a person present to help you set up and use this feature (**who?**). |
|  | Can you think of ways you could monitor your sedentary/non-sedentary activities in virtual reality and adjust it when necessary? | - An **activity** **logbook** showing you how active you have been in virtual reality.   - E.g., A video of a person, a virtual person or written information helping you to use virtual reality to set up and use this logbook.   - Have a person present to help you to set up and use the logbook (**who**) |
|  | Do you have any thoughts on the type of physical setting that would best support your use of virtual reality? (i.e., the room in which you put on the equipment and use virtual reality) | - Ensuring you have a **clear space** to use the virtual reality equipment. |
|  | Thinking about what you have learned about virtual reality today, can you think of ways other people could support you to be less sedentary using virtual reality? | - Have **other people committed** to taking part in non-sedentary virtual reality activities with you. - Have a **person present** to help you to set up and use virtual reality (**who?**). |
|  | Thinking about what you have learned about virtual reality today, can you think of ways virtual reality could help you or other retired/non-working adults believe the following things about themselves:   1. Identify as able-bodied and able to engage with virtual reality technology, not someone who is perceived as frail and unable to use this technology. 2. Believe that you are capable of being less sedentary and engaging with virtual reality. 3. Believe that being less sedentary will improve your future health. 4. Believe that being sedentary for prolonged periods of time each day could hinder your future health. | - A **reliable source** (**explore**) reassuring you that:   - You are able-bodied and able to engage with technology.   - You are capable of being non-sedentary by finding meaningful non-sedentary activities to take part in using virtual reality.   - Being non-sedentary by finding meaningful non-sedentary activities to take part in will improve your future health.   - Being sedentary for prolonged periods of time will hinder your future health outcomes.     - E.g., A video of a person, a virtual person or written information providing this information in virtual reality (1-4). - Ensuring the technology supports you to carry out tasks in virtual reality in a way that is **representative of your own ability** (body, movements, etc.) (1-2)   Are there any other instances or in any other forms/context you would like this reassurance? |
|  | Thinking about what you have learned about virtual reality today, can you think of what would motivate you to make the decision to be less sedentary using virtual reality? | - Provide **appealing non-sedentary virtual reality activities** to motivate you to decide to be less sedentary.   **Ask for suggestions on delivery of this content (i.e., what would make virtual reality appealing to them?)** |
|  | Can you think of ways you could set goals for the non-sedentary activities you complete in virtual reality? | - An **activity** **logbook** that allows you to **set goals** in virtual reality.   - A video of a person, a virtual person or written information helping you to use this virtual reality feature to set your goals.   - E.g., Have a person present to help you use this virtual reality feature to set your goals (**who?**). |
|  | Thinking about what you have learned about virtual reality today, can you think of meaningful non-sedentary activities you would look forward to that you could do in virtual reality? | - Take part in **enjoyable and meaningful** non-sedentary virtual reality activities with others or alone.   - A variety of appropriate virtual settings to take part in light-physical activity games – dance classes, golf, tennis, cycling, basketball, etc.     - What kind of activities would you like? |

**GENERAL QUESTIONS**

1. Is there anything else that comes to mind that you would include in this support system?
2. What do you think of the idea of healthcare practitioners providing the educational information?
   1. **Prompt:** Would you prefer it to be provided in a different way/by someone else?
3. Would you like to see the person providing the information embodied as an avatar or would a video work better?
4. Would you have any interest in using virtual reality to reduce your time spent sitting?
   1. **Prompt:** Any thoughts on why this is the case?
