# Supplementary material for: Exploring the content of the STAND-VR intervention: A qualitative interview study
Source: PLOS Digit Health. 2023 Mar 13;2(3):e0000210. doi: 10.1371/journal.pdig.0000210 (PMC10010507; doi:10.1371/journal.pdig.0000210)
Supplement: S2 Text — (DOCX) [file pdig.0000210.s002.docx]

**Demographic Questionnaire**

Please complete the following questionnaire.

*Note: Sedentary behaviour refers to any waking activity where minimal or no energy is exerted while in a sitting, lying or reclining position – e.g., driving or reading while sitting (activities such as cycling would not be considered sedentary behaviour as you are exerting significant energy while sitting).*

1. **Age (in years):** _________
2. **Sex**
   1. Male 🞏
   2. Female 🞏
   3. Prefer not to say 🞏
3. **Ethnicity (e.g., White Irish, Black Irish, Black African, Asian Irish, etc.)**

_____________________ Prefer not to say 🞏

1. **Are you permanently retired/not working?** Yes 🞏 No 🞏
   1. **Please specify how long you have been retired/not working?**

____________

- 1. **Please specify if you are retired OR not working:** _______________
  2. **If you are not working for reasons other than retirement, please specify the reason(s) you are not working?** Prefer not to say 🞏

Other: __________________________________________________

1. **Which of the following options best describes where you currently live?**
   1. Urban (in a city) 🞏
   2. Suburban (in a residential area surrounding a city) 🞏
   3. Rural town (in the countryside away from a city) 🞏
   4. Rural village (in the countryside away from a city) 🞏
   5. Rural area (in the countryside away from cities, towns, and villages) 🞏
   6. Prefer not to say 🞏
   7. Other: __________________________
2. **Highest level of education/qualification (e.g., primary, secondary, Bachelor’s, Master’s, PhD, qualified nurse, etc.):** ___________________
3. **Living Status**
   1. Alone 🞏
   2. With a Partner 🞏
   3. With a Family Member 🞏
   4. Other __________________

For each of the following activities, only count the time when this was your main activity. For example, if you are watching television and doing a crossword, count it as television time or crossword time but not as both.

***During a normal day****, how much time in total do you spend* ***sitting or lying down*** *while……*

| **SEDENTARY ITEM** | **TIME** | |
| --- | --- | --- |
| 1. Watching television or videos/DVDs | ______ hours | ______ minutes |
| 2. Using the computer/Internet | ______ hours | ______ minutes |
| 3. Reading | ______ hours | ______ minutes |
| 4. Socialising with friends or family | ______ hours | ______ minutes |
| 5. Driving or riding in a car, or time on public transport | ______ hours | ______ minutes |
| 6. Doing hobbies, e.g., craft, crosswords | ______ hours | ______ minutes |
| 7. Doing any other activities | ______ hours | ______ minutes |
