# Supplementary material for: Exploring the content of the STAND-VR intervention: A qualitative interview study
Source: PLOS Digit Health. 2023 Mar 13;2(3):e0000210. doi: 10.1371/journal.pdig.0000210 (PMC10010507; doi:10.1371/journal.pdig.0000210)
Supplement: S1 Table — (DOCX) [file pdig.0000210.s003.docx]

**IPAQ Data**

| **Participants** | **Time spent sedentary while watching TV** | **Time spent sedentary while using the computer/ internet** | **Time spent sedentary while reading** | **Time spent sedentary while socialising with friends** |
| --- | --- | --- | --- | --- |
| **PT001** | 3.5 | 0 | 1 | 1.5 |
| **PT002** | 3 | 3 | 0.5 | 0 |
| **PT003** | 5 | 3 | 0 | 1 |
| **PT004** | 3 | 3 | 2 | 2 |
| **PT005** | 4 | 1 | 2.5 | 2 |
| **PT006** | 2 | 0.5 | 1 | 3 |
| **PT007** | 2 | 1 | 1 | 2 |
| **PT008** | 2 | 3 | 1 | 2 |
| **PT009** | 3 | 0 | 1 | 2 |
| **PT010** | 4 | 2 | 1 | 0.5 |
| **PT011** | 6 | 0 | 2 | 0.5 |
| **PT012** | 3 | 1.5 | 2 | 2 |

| **Participants** | **Time spent sedentary while driving or riding in a car, or time on public transport** | **Time spent sedentary while doing hobbies, e.g., craft, crosswords** | **Time spent sedentary while doing any other activities** | **Total time spent sedentary each waking day (hours)** |
| --- | --- | --- | --- | --- |
| **PT001** | 1 | 1 | 1 | 9 |
| **PT002** | 1.5 | 0 | 0 | 8 |
| **PT003** | 0 | 0.5 | 2.5 | 12 |
| **PT004** | 0.5 | 0 | 0 | 10.5 |
| **PT005** | 0.5 | 0.5 | 2 | 12 |
| **PT006** | 0 | 1 | 2.5 | 10 |
| **PT007** | 1 | 2 | 0 | 9 |
| **PT008** | 1 | 2 | 1 | 12 |
| **PT009** | 2 | 1 | 1 | 10 |
| **PT010** | 1 | 3 | 2 | 13.5 |
| **PT011** | 0.5 | 0 | 0 | 9 |
| **PT012** | 2.5 | 0 | 1.5 | 12.5 |
