# Supplementary material for: Exploring the content of the STAND-VR intervention: A qualitative interview study
Source: PLOS Digit Health. 2023 Mar 13;2(3):e0000210. doi: 10.1371/journal.pdig.0000210 (PMC10010507; doi:10.1371/journal.pdig.0000210)
Supplement: S2 Table — (DOCX) [file pdig.0000210.s004.docx]

**Design Considerations**

| Accessibility | |
| --- | --- |
| Physical experience | |
|  | Consider including a virtual body to try and offset feelings of unsteadiness (only speculated by one participant) |
|  | Give IVR participants adequate time to get used to moving in IVR (5-10 minutes generally described by participants as enough time) |
|  | Ensure it is emphasized that reading glasses can be worn with IVR equipment |
| Head-mounted display | |
|  | Head-mounted display could be lighter |
|  | Ensure head-mounted display does not feel too tight on IVR participant |
| General accessibility considerations | |
|  | Consider enabling IVR equipment to connect to other digital devices |
|  | Consider making IVR hardware more visually appealing to older adults |
| Usability | |
| Learning preferences | |
|  | Offer options to learn how to use IVR in person |
|  | Have a person present to assist the participant – only needs to supervise rather than teach |
|  | No preference for the person providing the training |
|  | Prompt the participant when they are struggling with a task during their first experience (e.g., written pop-up instructions in the VE) |
|  | Have someone with experience with IVR teach participants how to use it for the first time |
|  | Provide instructions in non-technical language |
|  | Provide written instructions on how to interact with the VE |
|  | Happy to use instructions that come with product |
|  | Condense instruction manual (referring to instruction manuals in general) into something shorter and simpler |
|  | Talk through written notes with older people |
|  | Use written instructions in the VR FOUNDations VE in the future |
|  | Offer written instructions in the VE as well as a video as a video with instructions would disappear too quickly |
|  | Offer written instructions as well as an avatar providing instructions as they are more permanent than an avatar speaking and less distracting |
|  | Provide the option to have on call assistance |
|  | Provide feedback in the VE to make participants feel more comfortable |
|  | Provide feedback on participant performance in the VE |
|  | Provide encouragement to help participants get used to the equipment and the VE |
|  | Provide a combination of instructions to participants |
|  | Provide written instructions supplemented by a video depicting someone using the IVR equipment |
|  | Provide in person and written feedback |
|  | Blend sedentary behaviour information with IVR instructions (e.g., through IVR activity, you are being less sedentary) |
|  | Encourage participants to experiment with IVR themselves after their initial introduction |
|  | Give the choice to not engage with certain information provided in IVR |
|  | Provide an introduction with verbal and written instruction lasting 10 to 15 minutes |
|  | Offer IVR group learning sessions |
|  | Offer the choice to run IVR training sessions in a community organisation |
|  | Provide a graded learning experience |
|  | Offer the option to try IVR from a seated position for the first time |
|  | Begin with passive experiences and then move to interactive tasks |
|  | Build up to longer bouts in IVR over time |
|  | Organise any instructions in the VE in a way that enables participants to return to them at any time |
|  | Provide instructions directly from credible sources |
|  | Offer the choice to receive verbal instructions |
|  | Suggest trial and error as a way to get used to IVR |
|  | Consider explaining the IVR equipment before participants use it |
| Hand-Held Controllers | |
|  | Consider including more than one button function to increase choice of actions |
|  | Offer the choice to be able to see controllers in the VE |
| Interactions with objects | |
|  | Ensure participants are at a comfortable distance from the objects they interact with |
|  | Ensure written instructions in the VE do not get in the way of their line of view |
| Useful tools | |
|  | Offer the option to use a keyboard to interact with the VE where useful |
| User Experience | |
| Content preferences | |
|  | Make IVR more about practice and less about information gathering |
|  | Organise information provided in IVR into different stalls (i.e., like at a market) |
|  | Offer the choice to receive health information from an avatar |
|  | Deliver information provided by the avatar as a pre-recorded message |
|  | Offer the choice to receive health information from a virtual HCP avatar |
|  | Offer more interactive tasks in the VE as they are enabling |
|  | Offer a variety of activities in IVR to motivate participants to use it |
|  | Passive observation is not satisfying/engaging – need more interaction |
| Activity preferences | |
|  | Make IVR activities more engaging |
|  | Provide a variety of activities sought after by participants |
|  | Mountain climbing |
|  | Travel around the world |
|  | Cycling |
|  | Skiing |
|  | Tennis |
|  | Travelling back in time |
|  | Traveling to space |
|  | Painting |
|  | Fishing |
|  | Art activities |
|  | Bingo |
|  | Dancing |
|  | Gardening |
|  | Health and exercise |
|  | Cooking |
|  | Golf |
|  | Kayaking |
|  | Learning to sail a boat |
|  | Live stream of a play |
|  | Meditation and yoga |
|  | Quizzes |
|  | Seeing geological sites |
|  | Visit places of significance to participants |
|  | Provide competitive activities in IVR that can be done with other friends |
|  | Offer a combination of passive and interactive IVR activities |
|  | Organise IVR activities in order of physical intensity |
|  | Offer activities that involve movement |
| Avatar use | |
|  | Avatars are not necessary for individual experiences |
|  | Virtual hands could be more realistic |
|  | Avatar needs to appear to be a real person |
|  | Ensure social interactions in IVR can replicate the social cues that are present in in person interactions |
|  | Offer the choice to embody or not embody an avatar in IVR |
| Presence and immersion | |
|  | Avatar representation not necessary for some to feel present in the VE |
|  | Try to simulate more senses to make experience more immersive |
| Shared experiences | |
|  | Offer the choice to meet others in IVR |
|  | Offer the choice to share the IVR experience with others they know |
|  | Offer the choice to take part in group activities in IVR |
|  | Offer the choice to take part in live social physical activity events |
|  | Provide a virtual club to take part in virtual activities with others |
|  | Offer the choice to use IVR alone and with others |
| Safety features | |
|  | Make black and white feature of Oculus Quest 2 walk-through coloured |
| Resolution | |
|  | Improve crispness of the display |
|  | Make the graphics more realistic (similar to Oculus lobbies) |
| Exposure to IVR | |
|  | Include in instructions how long to spend in IVR |

Note. VE = virtual environment
