# Supplementary material for: Exploring the content of the STAND-VR intervention: A qualitative interview study
Source: PLOS Digit Health. 2023 Mar 13;2(3):e0000210. doi: 10.1371/journal.pdig.0000210 (PMC10010507; doi:10.1371/journal.pdig.0000210)
Supplement: S3 Table — (DOCX) [file pdig.0000210.s005.docx]

**Intervention Content Suggestions**

| **COM-B model component** | **TDF** | **Design Consideration** |
| --- | --- | --- |
| Physical capability | Physical skills | Limited technology experience is a barrier to use |
|  |  | IVR offers opportunities to take part in activities participants enjoyed in reality that they can no longer take part in due to physical limitations |
| Psychological capability | Cognitive and interpersonal skills | n/a |
|  | Knowledge | Preference to receive health information in a group setting |
|  |  | Lack of knowledge of digital technology is a barrier to use |
|  |  | Need to try IVR to understand what it is |
|  |  | Was not clear that full-scale environments could be experienced in IVR |
|  | Memory, attention and decision process | Developing a routine suggested as a strategy to use IVR |
|  |  | Prompts needed to remind some participants to use IVR at least until it becomes a habit |
|  | Behavioural regulation | Internal cue/mental note reminds them to be less sedentary |
|  |  | Preference to use physical calendar as a reminder (e.g., physical calendar, sticky notes) |
|  |  | Arrange time to use IVR every day |
|  |  | Do an IVR activity during TV ad |
|  |  | Gradually increase the use of IVR during the day over time |
|  |  | Preference to have the choice to do any activity rather than being reminded to do a specific one at a specific time |
|  |  | Open to mobile phone reminders to take part in IVR |
|  |  | Equipment could light up as a reminder |
|  |  | Provide activities that remind them to use IVR |
|  |  | Arranging to meet others in IVR a form of reminder |
|  |  | Would like timer to monitor their sedentary activities |
|  |  | Preference for on-screen reminders of how long they have been in IVR |
|  |  | Track progress over time but needs to be on their terms rather than being prompted over time |
|  |  | Timetable to monitor IVR activities |
|  |  | Offer choice to us an activity logbook in IVR |
| Physical opportunity | Environmental context and resources | Need to be introduced to IVR – not something you will happen upon yet |
| Social opportunity | Social influences | Community events suggested as the best approach to introducing retired and non-working adults to IVR |
|  |  | Retirement organisations offer opportunity to run issues by other members |
| Reflective motivation | Beliefs about capabilities | Trying VR will strengthen beliefs about capabilities |
|  |  | More motivated to use digital technology when it is made simple to use |
|  |  | Participants felt like they would not be able to use VR upon hearing instructions prior to use |
|  |  | Belief in capability established after a few practice sessions |
|  |  | Essential for IVR experience to be positive for it to strengthen beliefs about capabilities |
|  | Optimism | Needs to be positive outcome from using IVR |
|  | Beliefs about consequences | Motivator for using IVR is the benefit they would get from it by getting up and moving |
|  |  | Motivated to take part in activities in IVR that they can no longer do in the real world |
|  |  | Would be more motivated to use IVR if it helped prevent cognitive decline |
|  | Intentions | Would need training in how to use IVR before buying it |
|  |  | Needs to experience IVR before forming an opinion of it |
|  | Goals | Set daily goals to use IVR at a specific time |
|  |  | Reaching goal must be meaningful for individual |
|  |  | Preference to receive reminders of achievements |
|  |  | Likes idea of setting goals with others |
|  |  | No interest in activity log – appears to only need goals in the moment and not to keep track of activity |
|  |  | Taking part for the enjoyment is more important than setting goals |
|  |  | Would not see the benefit of setting goals to use VR unless it was recommended by a HCP |
|  | Professional/social role and identity | Frowned upon to be physically inactive in today's world when retired |
|  |  | Encouraging people to try IVR will help with identifying as someone who can use it |
|  |  | Control over virtual hands reinforced identity as someone who can use IVR |
|  |  | Repeated use needed to strengthen identity |
|  |  | Being older makes retired and non-working adults more aware of their vulnerabilities |
| Automatic motivation | Reinforcement | Preference to keep reminders of achievements to when they are in the environment |
|  | Emotion | Appeal to risky activities that can be facilitated in VR – underlying motivation to try these kind of activities |
