# Supplementary material for: Exploring the content of the STAND-VR intervention: A qualitative interview study
Source: PLOS Digit Health. 2023 Mar 13;2(3):e0000210. doi: 10.1371/journal.pdig.0000210 (PMC10010507; doi:10.1371/journal.pdig.0000210)
Supplement: S4 Table — (DOCX) [file pdig.0000210.s006.docx]

**Health Information Delivery Preferences Suggested by Participants**

| Avatar (ideally an HCP or other reliable source) providing health information |
| --- |
| Preference to receiving health information through a video rather than an avatar |
| Preference to receive health information on YouTube or google rather than in IVR |
| Usually uses the internet to access health information |
| Would be less distracted by written health information compared to a video |
| Preference to have live a consultation with HCP in IVR rather than pre-recorded session |
| Health information better to be delivered by friends – more supportive deliverer |
| Retirement groups could be a good source to provide health information |
| Would like to have group discussions with HCPs |
