# Supplementary material for: Exploring the content of the STAND-VR intervention: A qualitative interview study
Source: PLOS Digit Health. 2023 Mar 13;2(3):e0000210. doi: 10.1371/journal.pdig.0000210 (PMC10010507; doi:10.1371/journal.pdig.0000210)
Supplement: Attachment — Submitted filename: PLOS_Response_to_Reviewers.docx [file pdig.0000210.s007.docx]

Below is a record of the three additional references included in the revised manuscript to support changes made in response to reviewer comments. They appear below in the order they appear in the reference list. A table is also included with responses to each comment made by the reviewer.

8.	Kim Y, Welk GJ, Braun SI, Kang M. Extracting Objective Estimates of Sedentary Behavior from Accelerometer Data: Measurement Considerations for Surveillance and Research Applications. PLOS ONE. 2015 Feb 6;10(2):e0118078.

9.	Carson V, Wong SL, Winkler E, Healy GN, Colley RC, Tremblay MS. Patterns of sedentary time and cardiometabolic risk among Canadian adults. Preventive Medicine. 2014 Aug;65:23–7.

43.	Ekelund U, Tarp J, Fagerland MW, Johannessen JS, Hansen BH, Jefferis BJ, et al. Joint associations of accelerometer-measured physical activity and sedentary time with all-cause mortality: a harmonised meta-analysis in more than 44 000 middle-aged and older individuals. Br J Sports Med. 2020 Dec 1;54(24):1499–506.

| **Reviewer Feedback** | **Author Response (Extracts from revised manuscript)** |
| --- | --- |
| Author Summary p.3 “Prolonged sedentary behaviour, described as 6 or more hours…” I am personally unfamiliar with this definition of prolonged sedentary behaviour (SB). Prolonged sedentary time, usually refers to time spent in SB without breaks in sedentary time, i.e. sitting for more than 30/60min etc. | *Note. A detailed description of prolonged sedentary bouts is reported in the Introduction section*  Sedentary behaviour is defined as any waking behaviour that takes place in a sitting, lying, or reclining position each day while exerting little to no effort. Six or more hours of time spent sedentary each day has been associated with the development of a number of chronic conditions as well as mortality. Older populations tend to spend prolonged periods of time sedentary each day. |
| Introduction p.3 “Prolonged sedentary behaviour has been identified as a potential independent contributor to a number of chronic conditions as well as mortality (2–4).” See above.  p. 5 “This intervention content offers additional opportunities for retired and non-working adults to reduce the prolonged periods of time they spend sedentary each day using virtual reality.” In order to avoid confusion concerning the use of the word “prolonged” (see above prolonged SB), the authors could provide a brief definition of those prolonged bouts. | Prolonged sedentary behaviour has been identified as a potential independent contributor to a number of chronic conditions as well as mortality (2–4). Sedentary behaviour is defined as any waking behaviour which involves expending < 1.5 metabolic equivalents while in a sitting, lying or reclining position (5). Although clinical guidelines are yet to be established for prolonged sedentary behaviour, epidemiological evidence suggests that 6 or more hours of sedentary behaviour per day is associated with numerous morbidities and all-cause mortality as well as significant costs to public healthcare services (6,7). Prolonged sedentary behaviour can be defined in a number of different ways. For this study, we focused on what point a cumulative number of sedentary bouts, accrued throughout a single day, are correlated with negative health outcomes over time – which is approximately 6-hours (6). A sedentary bout can be described as a period of uninterrupted time spent in a sedentary position (i.e., sitting, lying or reclining while awake) (5). A conservative estimate of a single objectively measured sedentary bout is 10-minutes, with most sedentary behaviours “… observed within bout durations of <10 minutes…” (8). Carson and colleagues (9) specifically reported that ≥20-minute prolonged sedentary bouts could be particularly harmful to adults; with their study reporting associations between bouts of this length and higher insulin and lower diastolic blood pressure levels (p < 0.05), in a large sample of 4935 adults aged between 20–79 years. Other findings from this study suggested that each additional 10 breaks per day were associated with health outcomes such as higher HDL-cholesterol and lower insulin levels, among other outcomes. They concluded that breaking up these bouts throughout the day could mitigate negative health outcomes such as high insulin and low diastolic blood pressure. |
| Introduction  p.3-4 “epidemiological evidence suggests that 6 or more hours of sedentary behaviour per day is associated with numerous morbidities and all-cause mortality as well as significant costs to public healthcare services (6,7). Systematic review evidence indicates that many older adults are spending greater than 6 hours per day in a sedentary position (7,8), bringing them over the threshold for potential health risks that could impede their overall health and wellbeing into old age.” For readers of this manuscript, an estimate of older adults’ objectively monitored SB would be more informative here: self-report measurements typically drastically underestimate time spent in SB as stated by the review the authors cited (8). Harvey et al. 2015 found that "Objective measurement of SB shows that older adults spend an average of 9.4 hr a day sedentary, equating to 65-80% of their waking day. Self-report of SB is lower, with average weighted self-reports being 5.3 hr daily." | Systematic review evidence indicates that many older adults are spending greater than 6 hours per day in a sedentary position (7, 8) – with objectively measured sedentary activity specifically revealing that older adults are spending an average of 9.4 hours sedentary each day (8) – bringing them over the threshold for potential health risks that could impede their overall health and wellbeing into old age. |
| Discussion p.28 “With regards to certain misconceptions, some participants believed that the prolonged length of time they spend sedentary each day is not problematic as they believe they are exercising regularly outside of this activity. This same misconception, or distortion, can be seen in previous research (39), with systematic review evidence suggesting that this may be due to a lack of education regarding the effects of sedentary behaviour and how it differs from physical inactivity (40).” I disagree to some degree with the authors conclusion. Findings regarding this specific topic have been contradictory. More recently Ekelund and colleagues (2020) for example have found that the deleterious effects of sedentary behaviour might be attenuated by engaging in moderate to vigorous physical activity: “About 30–40 min of MVPA per day attenuate the association between sedentary time and risk of death, which is lower than previous estimates from self-reported data.” (Ekelund, U., Tarp, J., Fagerland, M. W., Johannessen, J. S., Hansen, B. H., Jefferis, B. J., . . . Lee, I.-M. (2020). Joint associations of accelero-meter measured physical activity and sedentary time with all-cause mortality: a harmonised meta-analysis in more than 44 000 middle-aged and older individuals. British Journal of Sports Medicine, 54(24), 1499-1506. doi:10.1136/bjsports-2020-103270) | The argument that moderate to vigorous physical activity cannot mitigate the negative health outcomes of prolonged sedentary activity alone has been contested to a degree in recent years, however, with a recent harmonised meta-analysis including more than 44,000 middle-aged and older adults reporting that “about 30–40 min of MVPA per day attenuate the association between sedentary time and risk of death…” (43). With this in mind, the information provided to people about sedentary behaviour must be considered further in light of this emerging evidence – emphasising the added importance of physical activity and the positive health outcomes associated with it. |
